# A complexity-informed in-depth case study into the sustainability and impact of a culture of health: The TR14ers community youth dance group

**DOI:** 10.1371/journal.pone.0293274

**Published:** 2023-10-25

**Authors:** Andrew James Williams, Katrina Wyatt, Kelly Stevens, Lisa Price

**Affiliations:** 1 Population and Behavioural Science, School of Medicine, University of St Andrews, St Andrews, Fife, United Kingdom; 2 Department of Community and Health Sciences, Exeter Medical School, University of Exeter, Exeter, Devon, United Kingdom; 3 Environment and Sustainability Institute, University of Exeter, Penryn, Cornwall, United Kingdom; 4 Department of Sports Science and Public Health, University of Exeter, Exeter, Devon, United Kingdom; University of Ghana, GHANA

## Abstract

There are calls for researchers to study existing community assets and activities that appear to improve health and have achieved longevity. The TR14ers Community Dance Charity Limited is a community youth dance group that has been running since 2005 providing free weekly sessions for children and adolescents in an economically disadvantaged town in the UK. An in-depth case study employing qualitative, quantitative and participatory methods was undertaken with the TR14ers (current participants and those who have left, co-ordinators and families) over 6 months with the aim of understanding the sustainable processes and impact of the Group. The 12 complex systems’ leverage points described by Meadows and the five domains of adolescent wellbeing developed by the United Nations H6+ Technical Working Group on Adolescent Health and Well-Being were used as frameworks to recognise the complexity of community assets like the TR14ers. The quantitative and qualitative data indicated that being part of the TR14ers contributed to multiple health and wellbeing outcomes. The positive experiences of being a TR14er led members to actively recruit others through word of mouth and public performances. Central to the TR14ers is a commitment to children’s rights, which is communicated formally and informally throughout the membership informing how and what the Group does, leading to the structure and delivery of the Group evolving over time. Members sought to ensure the sustainability of the Group after they had left and were keen to mentor younger members to develop and become the leaders. Based on the insights from this case study we suggest that efforts to develop cultures of health, like the TR14ers, should focus on the core values of the activity or intervention that underpin what it does and how within the local context.

## Introduction

Non-communicable diseases are now the leading cause of disease and death around the world [1]. The World Health Organization [1] identify four behaviours as significantly increasing the risk of non-communicable disease: tobacco use, physical inactivity, the harmful use of alcohol and unhealthy diets. However, these four behaviours are shaped by a wide set of socioeconomic, commercial, cultural and environmental determinants [2]. These determinants differ between countries and communities, having different impacts on different populations generating inequalities in morbidity and mortality [3,4]. This makes it difficult to develop interventions with universal effectiveness.

There are a wide range of evidence-based programmes that aim to increase population physical activity [5,6]. However, evidence suggests that many of these interventions exacerbate inequalities for individuals with key protected characteristics or other vulnerabilities, low socioeconomic status or geographic deprivation [7,8]. Interventions not only need to produce a meaningful effect on physical activity, but also sustain the effect. There are increasing calls for practice-based evidence whereby we learn from ‘successful’ practices already taking place, including community assets, to inform commissioning and research [9–13]. However, undertaking evaluative research into community-based activities is often challenging as they are embedded in complex adaptive social systems and therefore cannot be controlled to the extent necessary for a randomised controlled trial evaluation. Greenhalgh and Papoutsi [10] in writing about complex interventions suggested that:

‘we need research designs and methods that foreground dynamic interactions and emergence—most notably, in-depth, mixed-method case studies that can act as concrete, context-dependent exemplars, including powerful ethnographic narratives paying attention to interconnectedness and incorporating an understanding of how systems come together as a whole from different perspectives.’

[10, p.2]

Our aim was to conduct such an in-depth, mixed-method case study to understand the sustainable processes and impact of engaging young people in a peer-led dance group: the TR14ers [14]. The TR14ers are a community youth dance group, formed in 2005, who have created an inclusive environment that engages young people experiencing economic inequalities in weekly street dance sessions and performances. Understanding the process by which the Group engages young people, as well as the processes underpinning the sustainability of the Group will provide much needed evidence for commissioners, practitioners and researchers seeking to commission, deliver and develop sustainable health creating activities. The four objectives of the whole study were:

1. to collect process data to understand how and why young people participate in the TR14ers
2. to co-develop a logic model that accurately captures the processes of engagement and participation in the Group and hypothesised short-and long-term outcomes
3. to co-develop valid, feasible and acceptable methods for collecting baseline process and outcome data on a sufficient number of TR14ers
4. to co-develop a full evaluation proposal; including identifying counterfactuals and routinely collected datasets for comparison

Objectives three and four were related to developing a full evaluation of the TR14ers to follow-on from the case study and therefore this paper is focused on objectives 1 and 2.

In order to ground the research in existing thinking about complex systems and adolescent health two existing frameworks were used to support the interpretation of the data. The environmental and systems scientist Meadows [15] outlined the following 12 complex systems’ leverage points:

1. Transcending Paradigms—The power to transcend paradigms
2. Paradigms—The mind-set out of which the system—its goals, structure, rules, delays, parameters—arises
3. Goals—The purpose or function of the system
4. Self-organisation—The power to add, change, or evolve system structure
5. Rules—Incentives, punishments, constraints
6. Information flows—The structure of who does and does not have access to information
7. Reinforcing feedback loops—The strength of the gain of driving loops
8. Balancing feedback loops—The strengths of the feedbacks relative to the impacts they are trying to correct
9. Delays—The lengths of time relative to the rates of system changes
10. Stock and flow structures—Physical systems and their nodes of intersection
11. Buffers—The sizes of stabilizing stocks relatives to their flows
12. Numbers—Constants and parameters such as subsidies, taxes, standards

These 12 leverage points have recently been translated to encourage their use in public health research and practice by Bolton, Whelan [16], to understand how change can happen in complex systems. The second framework was the five domains of adolescent wellbeing developed by the United Nations H6+ Technical Working Group on Adolescent Health and Well-Being [17]. This was selected to provide a broad understanding of health for adolescents who are less likely to have a disease, but still experience variations in health. The five domains include physical, mental and social wellbeing but also environment, skills development and autonomy [17].

### Background to the TR14ers community dance charity limited

The TR14ers Community Dance Charity Limited is a peer-led dance group in West Cornwall, United Kingdom [18]. The Group was initially formed by the town’s police and young people in 2005 through the Connecting Communities (C2) Programme [19] in response to persistent high levels of anti-social youth behaviour. The C2 Programme explicitly embraces the complexity of social systems; recognising interdependencies between residents and service providers and creating feedback loops to support learning and sustain new relationships to enable health and wellbeing. C2 has been recognised by the National Institute for Health and Care Excellence and Public Health England as an example of transformative community development [19,20]. The police having recognised that handing out more and more anti-social behavioural orders was having no impact on behaviour; walked around the town asking young people ‘what is it like to live in the town?’ and ‘what activities would they like to engage with?’. The young people identified dance as an activity they would engage with and the initial dance sessions were held in the school holidays, in a local nightclub, supported by a dance choreographer. The name TR14ers was based on the area’s postcode as the young people did not want the town’s name in the title; feeling there was a stigma attached to being from Camborne. Since 2005, it is estimated that over 3,000 young people have been part of TR14ers, with current membership extending beyond the TR14 postcode area. Members having gone into dance training, youth work, higher education and employment nationally as well as locally.

The Group receives no formal funding and is reliant on awards from charities, local organisations and individual donations. Membership of the Group is free, as is transport to public performances and TR14er branded T-shirts. At the time of the study the Group held weekly dance sessions, and performed at various events throughout the year. Between 40 and 60 young people attended each weekly sessions, and while there are Co-ordinators and volunteers (crew), instruction was provided by dance leaders who were members of the Group. There was a leadership programme with those aged 10 years or older able to become Recruit and then Elite leaders. A member of the TR14ers will either volunteer or be approached about becoming a leader. They will initially become a Recruit leader that means they take part in similar activities and roles as the Elite leaders but are still developing their leadership skills. After a period of months or years as a Recruit leader they can then ask to become an Elite leader, this triggers a period when they are observed to check that they are demonstrating the characteristics and values required to become an Elite leader. There is a contract signed by the Recruit and Elite leaders that outlines the expectations of them (S1 Appendix). The weekly dance sessions last 2 hours after school on Friday evenings. There is a warm up at the beginning and cool down at the end of each session, and in between these activities the Recruit and Elite leaders teach and rehearse routines in preparation for the next performance. The whole group may learn a routine together or they can split into smaller groups for separate routines, and there are regular refreshment breaks. After each session the Recruit and Elite leaders stay for a further hour to prepare for the following week and develop their teaching skills, with the Elite leaders remaining for one further hour of rehearsal and preparation. While on the surface the TR14ers provide young people with the opportunity for up to two-hours of moderate to vigorous physical activity a week; talking to the young people and observing one of their weekly dance sessions quickly reveals the highly complex nature of the ‘intervention’ as well as the multiple, interacting systems in which the Group are embedded [21–23].

There is an extensive body of literature (including dedicated journals) on community dance, including the use of community dance for a variety of health and social needs. However, the focus of this study is on the application of a complexity-informed approach to understand the process by which a community health asset engages young people, as well as the processes underpinning the sustainability of the Group.

## Methods

With the ambition of applying for funding to undertake a full evaluation of the TR14ers, the process we sought to undertake with Rapid Funding Scheme funding was an evaluability assessment [24]. This is described as a ‘pre-evaluation activity designed to maximize the chances that any subsequent evaluation of programs, practices, or policies will result in useful information’ [24, p.214]. A critical phase of an evaluability assessment is getting a shared understanding of the realities of any intervention, usually through producing a logic model or programme theory. Subsequently, the evaluability assessment process offered a process for meeting our aim and objectives around understanding how TR14ers engage young people and maintain that engagement in a culture of health [25].

A preliminary logic model for the TR14ers was developed from the information contained in the annual reports and website. This logic model (S1 Appendix) developed prior to data collection; illustrated the inputs, outputs and outcomes reported for the group in their documentation, but did not contain much information about the activities. Therefore, the approach adopted in this study was to use the types of in-depth mixed methods Greenhalgh and Papoutsi [10] and Paparini, Green [14] have indicated are necessary in order to test and refine the logic model into a programme theory. The qualitative methods included observations of the weekly dance sessions and interviews with Co-ordinators, past members (alumni) of the Group and parents of current members. The quantitative methods focused on current members and included: (i) a review of the attendance logs taken at each weekly dance session to understand patterns of engagement and (ii) trialling of methods to collect possible outcome data including questionnaires and activity data (accelerometer). We also held two co-development workshops with current TR14ers; the first of which was to refine the logic model and the second was to agree key outcome measures and methods for collecting them.

The study took place in Camborne between October 2018 and March 2019 and participants were recruited throughout this period. The study was approved by the University of Exeter Medical School Ethics committee (reference number: Oct18/B/184). The ethics approval required that written informed parental/legal guardian consent and child assent was collected for any participant aged less than 14 years, while those aged over 14 years provided their own written informed consent. The study was pre-registered at https://www.isrctn.com/ISRCTN15513557 on 31 August 2018. The Co-ordinators, parents and current members were recruited during the weekly sessions. Parents who were either involved as helpers (the crew) during the sessions or stayed outside the session while their child participated were invited to be interviewed. Alumni were contacted through the wider network of TR14ers including older siblings of current members. Age appropriate participant information sheets and consent forms, as well as the opportunity to ask any questions were provided. For practical reasons current members needed to have attended at least 4 of the weekly TR14ers sessions during the study to participate, which gave time for consent forms to be sent home and returned to the researchers as well as any data collection processes.

### Qualitative data collection and analysis

Researchers attended most of the weekly TR14ers sessions during the 6 month study period. As well as collecting the quantitative data described below, they undertook observations of the dance sessions, the interactions between members, leaders and helpers and recorded their reflections on each session. These field notes were used to help address our first two research objectives. The observations of the weekly dance sessions were the primary method for learning about the activities of the TR14ers.

Seeking to learn about how people initially engaged with the TR14ers and how the group has been sustained over so many years, three groups of participants were invited to take part in interviews: the co-ordinators, alumni (former members) and parents of current TR14ers. KS undertook the interviews using a topic guide (S2 Appendix), and all the interviews were audio recorded and transcribed for analysis. In total 13 interviews were conducted: six parents of current TR14ers, five TR14er alumni, and the two TR14er Co-ordinators. On average these lasted 45 minutes with one 30 minute interview and two exceeding 1 hour. The transcripts were de-identified and shared between KS, KW and AJW using a secure university file sharing platform for thematic analysis. The original recordings were destroyed once the transcripts had been anonymised and confirmed as accurate.

There were three areas of focus for the thematic analysis. Firstly, what outcomes (short and long term) were reported to be associated with TR14er participation? This was informed by the five domains of adolescent wellbeing developed by the United Nations H6+ Technical Working Group on Adolescent Health and Well-Being [17]. It was felt that the final interview question (S2 Appendix) was leading and therefore responses to that question were removed from the analysis. Secondly, how did people learn about the TR14ers and start participating? The responses to the fourth question in the topic guide (S2 Appendix) were coded to address this question. Finally, we sought to understand the sustainability of the group particularly through the application of 12 complex systems’ leverage points [15]. Themes were coded and cross-checked by KS, AJW and KW to ensure consistency in data interpretation. Field data from observations and informal conversations were triangulated with the in-depth interview data. Importantly these findings were fed back to the participants in the co-development workshops to check their credibility with the experience of the young people who were current members.

### Pilot quantitative data collection and analysis

The outcome measures to be quantitatively pilot tested were based on the preliminary logic model (S3 Appendix). Due to the wide age range of the TR14ers it was necessary to collect different outcome measures or collect them differently depending on the age of the participant. The database of outcome and experience measures curated by the Child Outcomes Research Consortium [26] was used to identify age-appropriate instruments for the collection of the outcome data. The Child Outcomes Research Consortium [26] is ‘the UK’s leading membership organisation that collects and uses evidence to improve children and young people’s mental health and wellbeing.’ The following outcomes and instruments were selected for testing in this study.

1. Physical activity assessed using GENEActiv [27] wrist worn accelerometers for one week (85.7 Hz measurement frequency, 24 hours a day)
2. Health Related Quality of Life assessed using the validated self-completion KINDL instruments [28]
3. Self-esteem using the Adolescent Self-Esteem Questionnaire [29] [≥11 year olds only]
4. Smoking and alcohol consumption using the questions from the Health Survey for England [30] [≥11 year olds only]
5. Diet using the food intake questionnaire (FIQ) [31,32] [≥9 year olds only]
6. Nutrition knowledge tests based on the United Nations Food and Agriculture Organisation (UN FAO) guidelines [33] [≥11 year olds only]

Our primary intention was to test the feasibility and acceptability of collecting these data. However, national data were available for outcomes a-d, making it possible to undertake some simple comparisons between the TR14ers and these other datasets. The analyses of these variables were purely descriptive and the data were anonymised once accurate entry had been confirmed.

### Co-development workshops

The first co-development workshop was held in October 2018 during the school half term holidays. The purpose of the first workshop was to share and refine the preliminary logic model of how we understood the TR14ers (S3 Appendix) with current members. The workshop activities included the young people creating and acting out adverts for the TR14ers (to understand what they thought being a TR14er meant and its impact on them) and using magazine clippings or drawings to illustrate what they associated with being a TR14er and any changes in their lives they felt being a TR14er had created. The intended output of this workshop was a revised version of the logic model to begin to identify outcome measures and possible areas to explore in the interviews, and therefore only amendments and additions to the logic model were recorded from this workshop.

The second co-development workshop focused on possible designs for the full evaluation and therefore only Elite and Recruit leaders were invited. This was held during the school half term holiday in February 2019. This was an opportunity to gather feedback on their experiences of completing the quantitative data collection and discuss what they felt was feasible and acceptable in terms of how often the data were collected and how best to collect it. The intended output of the workshop was agreement around a set of outcome measures and the frequency of data collection for the full evaluation proposal.

### Research team

There were four members of the research team. KW led the project and is a Professor of Relational Health with significant experience of evaluating complex interventions and part of the C2 programme; she had also worked with the TR14ers previously [19]. AJW was a lecturer in public health at the time and co-investigator on the grant. He has significant experience evaluating complex interventions and conducting evaluability assessments. KS was employed as the research associate on this project; she is an experienced qualitative researcher with a background in narrative methods. This was the first time both KS and AJW had worked with the TR14ers; although AJW had been aware of them for a number of years. LP processed the accelerometery data and the research team was supported with the project record keeping, data management and administration by a Data Manager.

## Results

**Fig 1:**
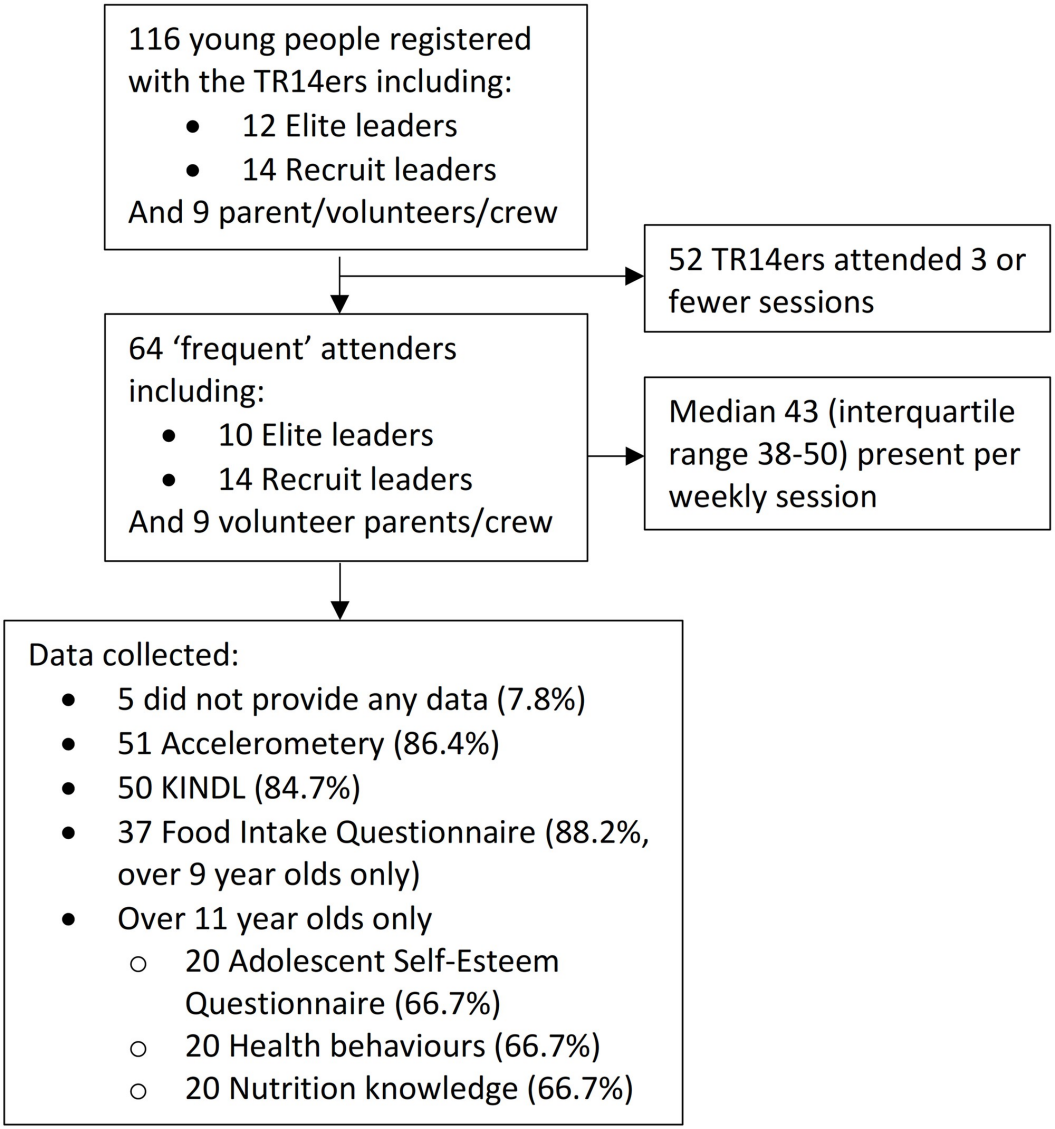
TR14ers baseline study flow diagram (September 2018-March 2019).

Participation of current TR14ers in the weekly dance sessions and the study during the study period are summarised in Fig 1. Of the 64 young people who participated in four or more of the weekly dance sessions (based on the attendance logs) during the 6 months, 59 (92.2%) participated in at least one aspect of the study. The participants included some who had joined during the study period and others who had been members for 8 years. The mean age of participants was 11.0 years (standard deviation 3.2 years) and 81.4% of participants were female. Valid postcodes were provided by 57 of the 59, 22% of which were from non-TR14 postcode areas. In terms of area-level deprivation, most participants were from decile 4 of the Index of Multiple Deprivation (IMD, range 1–7). With the participants keen to demonstrate the benefits of the TR14ers, we had high compliance with the quantitative data collection, especially the accelerometery (85% of participants wore the accelerometers for 8 days, 24 hours a day). Through the use of age-appropriate questionnaires (administered during breaks in the dance sessions) there were few issues encountered with collecting these data.

Twenty five young people who were current members of the TR14ers participated in the first co-development workshop and half of the current Elite and Recruit leaders (n = 12) participated in the second co-development workshop. During the first co-development workshop the young people confirmed that the outcomes we identified were appropriate. However, based on their comments about what being a TR14er meant to them, and the clear link they made between participation and self-esteem, the Australian Adolescent Self-Esteem questionnaire was added [29]. Following the first co-development workshop the preliminary logic model (S3 Appendix) was updated. During the second co-development workshop we agreed a study design for a full evaluation of the Group that was developed into a grant and submitted for funding.

The pilot findings in relation to each outcome are described in the openly available final report to the funders [34]; the outcome data are summarised below, however the main focus of this paper is how TR14ers engage young people and the processes by which they sustain engagement in the Group.

### Summary of reported outcomes

The qualitative data from TR14er alumni and parents provided rich insights into the wide number of physical, mental, emotional and social health and wellbeing outcomes, reportedly associated with participating in the Group in the short, medium and long term. In S4 Appendix we have provided data and quotes that illustrate how the TR14ers map to the five domains of adolescent wellbeing developed by the United Nations H6+ Technical Working Group on Adolescent Health and Well-Being [17]. These qualitative findings were supported by the findings for the quantitative outcomes that were particularly notable in the context of a low-income community. Only two (10%) of those completing the Adolescent Self-Esteem questionnaire had a score ≤17 that had previously been found to be indicative of a greater than 50% probability of having depression [29]. Smoking and alcohol consumption experiences were similar to those reported in the nationally representative Health Survey for England [35]. Over 60% of the young people undertook 30mins or more moderate-to-vigorous physical activity (MVPA) on 5 or more days of the week, whereas the Health Survey for England had only identified 20% of young people achieving this level of activity [36, data accessed through NESSTAR]. Although, it is possible that this difference compared with the Health Survey for England data may have arisen from the use of wrist worn rather than hip worn accelerometers or different thresholds or units for calculating moderate to vigorous physical activity [37,38]. As pilot data, these outcomes cannot be solely attributed to participation in the TR14ers, however, these initial indications of beneficial outcomes demonstrate the need for this case study to understand how the TR14ers have formed and maintained a culture of health.

**Fig 2:**
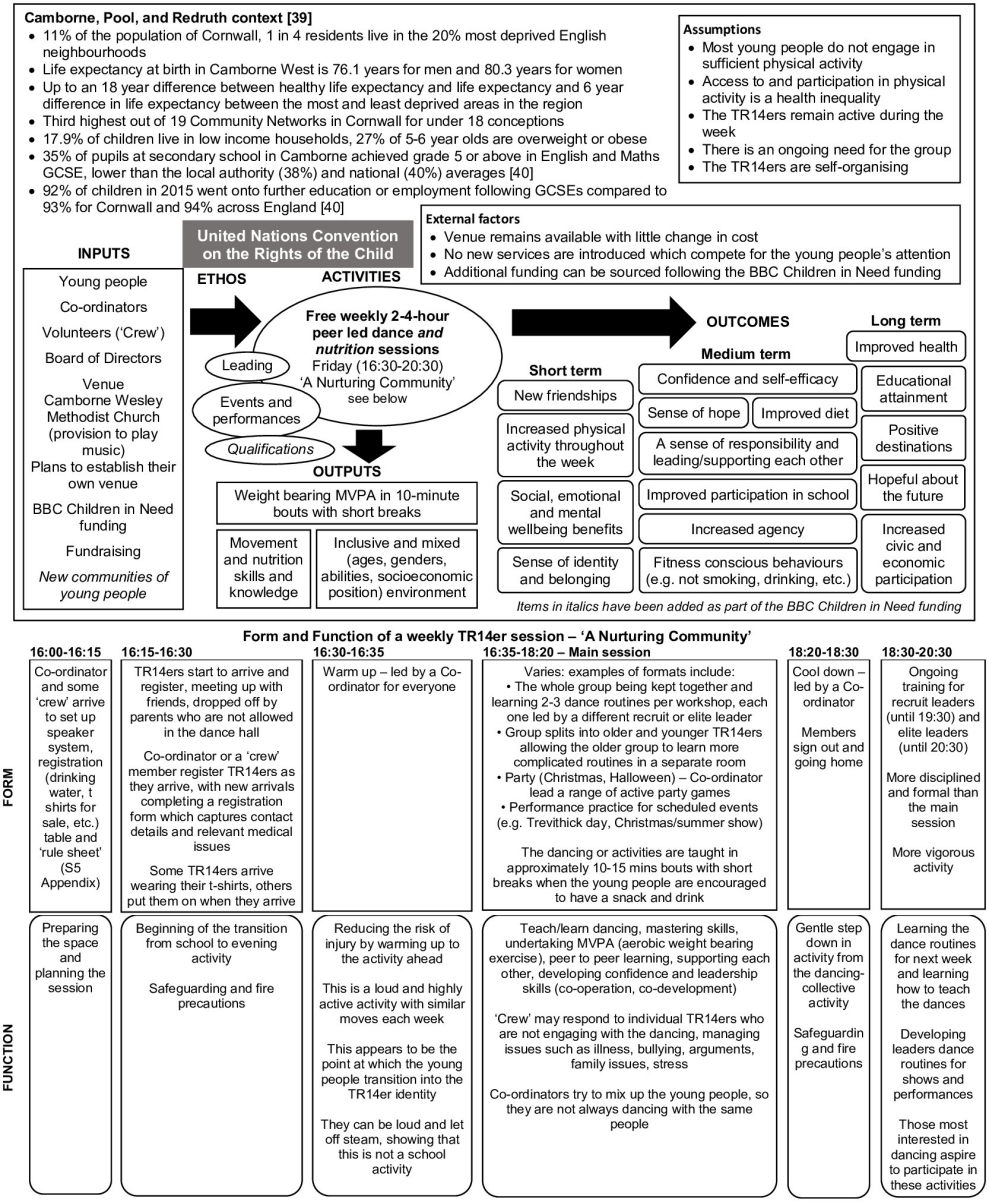
TR14ers two part programme theory.

Synthesising the qualitative and quantitative date allowed us to generate a two part programme theory (Fig 2) for the TR14ers: the first part of which documented the context, inputs, outputs and outcomes of the intervention, while the second part described the intervention activities in terms of form and function [39,40]. We also observed formal terms of engagement like a poster of rules or attributes of TR14ers displayed during the dance sessions (S5 Appendix) and the Leader’s Contract (S1 Appendix) as well as informal methods of engagement the group fosters.

### How young people hear about the TR14ers

All five of the TR14er alumni and five of the six parents interviewed described learning about TR14ers through word of mouth. The two quotes below demonstrate that those involved with TR14ers want to encourage others to join.

‘So actually, I was at Butlin’s [a UK holiday resort] a few years ago and there were people from Cornwall up there and there was a young girl with really bad anxiety, really bad. And I even said to her about coming along. I said to her, “Look, you’re not far away.” Redruth, they were from. “You’re not far away, go to TR14ers.” And they did for a while.’

(Alumni 4)

‘I’ve sold it to a few people [laughs]. I’ve managed to get a few people, three or four children to come.’

(Parent 5)

The only other method of learning about the TR14ers was seeing a public performance. While neither of these are techniques commonly used to recruit into public health interventions; they provide two important insights into the process of engagement with this culture of health. Firstly, that personal experience or recommendation prompted people to join the group. Secondly, the young people enjoy TR14ers and want to recruit new members. This second point starts to address why the TR14ers have been sustained over so many years.

### How the TR14ers have been sustained over so many years

The following series of quotes from Alumni 2 illustrate the process, echoed over a number of the interviews that was reported to have sustained the TR14ers since 2005.

‘It’s a culture set by the leaders … and whoever’s been in charge. It’s evolved through what we learn has gone well and what hasn’t gone well, they just have a really good—I don’t know what the word is—they just show a lot of compassion to the young people and they feel like, they are like, they can get their voice heard if they want to say something, and they can just feel free to do and say what they need to say without being restricted.’

‘Yeah. It works its way down, from the people who lead it, the people in charge through the leaders especially, and then the kids follow that, then it kind of builds up from that, so the leaders will mentor as such, naturally, without realising, because it’s what their leaders are doing.’

‘Yes because you want your next batch of leaders, your next group of leaders to take over once you’ve left, so you want to mentor and get the best, if you want TR14ers to continue when you’re too old, when you want to move on, then you’re going to want to have the best people leading it as you can.’

(Alumni 2)

The focus on creating supportive, inclusive relationships to engage young people in dance, rather than dance as an end in itself as well as the ’rules’ underpinning engagement and participation show how the TR14ers operate as an open, dynamic system, adaptive to the needs of its members and the wider environment. These are characteristics of a system that is complex, which was further illustrated through mapping the findings from the observations and interviews to the 12 complex systems’ leverage points (Table 1) [15].

**Table 1:** Mapping TR14ers to the Meadows [15] 12 complex systems’ leverage points and Public health 12 framework [16].

	Meadows 12 complex systems’ leverage points	Public Health 12 (PH12)	TR14ers evidence
1	Transcending Paradigms—The power to transcend paradigms	The ability to continually adapt collective fundamental beliefs leading to widespread change in the way things are, to respond effectively to multiple complex problems.	This leverage point is around spreading the paradigm and practice to other surrounding systems. The TR14ers have not attempted to alter the systems surrounding them; although in smaller ways the members demonstrate how their experience of the TR14ers shapes how they interact with others in their education, training or employment.
2	Paradigms—The mind-set out of which the system—its goals, structure, rules, delays, parameters—arises	A population-level shift in fundamental beliefs (e.g. cultural shift) on how to respond effectively to complex problems (a change in the way things are).	All children have rights and poor health, wellbeing and inequalities arise from lack of access to those rights. The United Nations Convention on the Rights of the Child [41] ‘I’m incredibly passionate that they are such formative years and if they are having a really tough time outside of there, on a Friday that’s their safe place and they feel that that is their safe place, and they can grow in that time, they can learn in that time, then I’m happy.’ (Co-ordinator 1) ‘…there’s got to be a core ethos that every single person in that room is equal to the other; there’s no-one better than the other.’ (Co-ordinator 1) ‘TR14ers is all about making sure that you are part of the family on the team but everybody is still individual inside it, so it’s not like we are saying, “Right, you have to teach like this and you have to teach like that,” it’s they want to teach the way they teach, so we help them express that. It’s not that we are telling them they have to do a specific style, they want to do what they want to do, we help them adapt.’ (Alumni 3)
3	Goals—The purpose or function of the system	Where a fundamental goal of a system is challenged and changed.	Children who have access to their rights. Children who are needed ‘Empowerment through dance: making a positive difference to young people’s lives’ [18]
4	Self-organisation—The power to add, change, or evolve system structure	Creating and maintaining infrastructure (e.g. political or governance) for implementing a combination of various level 5–12 actions over time.	Leadership programme and role modelling of behaviours (S2 and S3 Appendices) ‘…if they tell you something and then they see it happen within the next two weeks, then they know. So if they say, “We really need an extra room because the older dance teachers, the leaders, need somewhere to get down and talk before they go in. There’s a room next-door; can we have that?” And then they get that room, then they’ve made an executive decision, it’s been discussed, it’s happened and the treasurer has paid the money.’ (Co-ordinator 2) ‘I think you need that change every now and then. Without change it gets so boring.’ ‘So yes, you always need to change every now and then just so it just keeps that loop of, okay, there’s something new going on, so it keeps it alive.’ (Alumni 5)
5	Rules—Incentives, punishments, constraints	New modified rules such as incentives and accountability mechanisms for change.	Terms of engagement (S1 and S5 Appendices) ‘So what do you think people get out of TRs? … “They have punctuality, certain times you’ve got to be there. Responsibilities, there’s quite a few rules people need to stick to.”‘ (Alumni 1)
6	Information flows—The structure of who does and does not have access to information	Movement of vital information to shift power dynamics that opens the decision-making processes to more (and the right) people.	The approach to information and information flows within TR14ers are especially open and transparent. There is shared responsibility and a sense of reciprocity in looking out for each other. Information can flow rapidly resulting in small adjustments through to proposals at the Board of Directors. ‘One thing we don’t do is we don’t count them off. We don’t count off their ages, we don’t count off their gender and we don’t count off their sexuality or their race or any of that stuff. It’s free and they can come.’ (Co-ordinator 2) ‘Yes, because you want them to feel like they have ownership of things like the way TRs helped me feel like I had ownership of, we all had an ownership of, our dance group. I kind of want the younger people, this generation that I’m leading now, to know that this is, they have ownership, this is for them.’ (Alumni 2)
7	Reinforcing feedback loops—The strength of the gain of driving loops	Initiating a movement toward a target that is self-reinforcing and growing exponentially in the desired direction.	Leadership programme and membership of Board of Directors—role modelling responsibility and reciprocity ‘So I was in the board meetings, I got to be really involved with organising fundraisers, all sorts of things that she got me having, just gave me loads of responsibility basically so that helped me understand how things work and that gave me more confidence in how to teach and lead my classes a bit more’ (Alumni 2) ‘and if TR14ers can help me be able to speak to people which sounds really small from going to speaking to no one and really downhilling to coming back up again and doing everything that I’m doing now then yes, I think it can help a lot more people. It just needs to keep going.’ (Alumni 2)
8	Balancing feedback loops—The strengths of the feedbacks relative to the impacts they are trying to correct	Taking action to stabilise a part of the system to achieve a specific intended goal.	Leadership programme and membership of Board of Directors, again shared responsibility for maintaining the TRs ‘It’s the whole if one of us is failing then we are all failing because we haven’t picked up on it, so if one person is upset or stressed out or something then it falls on the rest of us to make sure that we pick up their slack.’ … ‘I think that’s what’s rubbed off on the rest of the TRs, is the fact that everybody will put themselves on the backburner to make sure that everybody else is okay and that is like the main focus of what the TRs is.’ (Alumni 3)
9	Delays—The lengths of time relative to the rates of system changes	Strategic planning to align timeframes with available resources, current readiness, and intended outcomes.	Ability to trial and evolve at the pace of the young people, meaning that TRs maintains some recognisable components, but has also changed over time. The shared responsibility for TR14ers means that ideas can be suggested, but it is understood that these might need to wait until funding becomes available. This shapes some of the TR14ers activities to the timelines of funders.
10	Stock and flow structures—Physical systems and their nodes of intersection	Building of new physical infrastructure, providing financial infrastructure, and/or improving physical movement through the system.	The availability of a venue and funding are the key stocks. The group has plans to become income generating (self-sufficient) through owning their own venue. Having their own venue would also contribute to the identity of the TR14ers and sense of belonging in Camborne. ‘If you’re not charging people to do it, where are you going to get the money from? To the stage where there was actually a time when me and [Another alumni] were ready to pay for the hall out of our own wages, a few years ago. We really were, we were ready to do it, but thankfully [Co-ordinator 1] got the funding in from somewhere—or [Co-ordinator 2] did. Both of them did. But yes, I’ve seen it from its all-time high to where it was all-time low and now, where it’s on the rise again. But I love this place.’ (Alumni 4)
11	Buffers—The sizes of stabilizing stocks relatives to their flows	To maintain a safety net within our community or system to absorb reasonably foreseeable, but unexpected events without adversely affecting the way things are. This includes supports for individuals and groups built into environments, schools, workplaces.	Maintaining financial reserves and being able to adapt the scale and activities of the Group without deviating from the fundamental beliefs.
12	Numbers—Constants and parameters such as subsidies, taxes, standards	To increase or decrease one isolated, existing part of the system.	No barriers to participation (i.e. free), the venues available and the facilities at those venues, recruitment through events and word of mouth ‘…the real advantage of it being free is, hey presto, the kids take ownership of it. Because if they haven’t got to pay a fee to walk in the door, the feeling is it must be theirs. So that’s why we get all this leadership going on, because they naturally become leaders’ (Co-ordinator 2)

The highest of the 12 leverage points at which it was possible to map findings from the TR14ers was point 2, the paradigm; ‘the mind-set out of which the system—its goals, structure, rules, delays, parameters—arises’ [16, p.3]. The three illustrative quotes mapped to the paradigm in Table 1 mention safety, happiness, equality, support, independence and freedom of expression all of which are children’s rights; formalised in the United Nations Convention of the Rights of the Child [41]. This paradigm, the commitment to children’s rights, was evident through the use of language and nature of the relationships observed. It was communicated formally (terms of engagement, S1 Appendix and S5 Appendix) and informally (thought actions and role modelling) to all members, especially through the leadership programme, shaping what the TR14ers do and how. Reinforcing and balancing feedback loops keep the TR14ers adapting and developing so that one alumni said ‘It’s different and the same … I love how it grows; throughout the year’ (Alumni 2).

Significantly, and similar to the desire to invite other people to join; the members want and recognise the need for the Group to continue after they leave. The members therefore want to develop others who can take on leadership and keep the Group going. Other quotes in Table 1 and S4 Appendix illustrate that there have been challenges and periods of growth and loss during the 18 years, including a period when the members were contemplating paying the rent for the room themselves.

‘it sounds really corny and cheesy but it really is a family. You come, you learn, you go and, over the years, you go on to better things’

(Alumni 4)

## Discussion

This mixed methods case study analysing and interpreting an existing and long sustained community dance intervention for young people through the lens of complexity science and mapping the findings onto the 12 complex systems’ leverage points from Meadows [15], has identified the mechanisms by which the activity engages young people and sustains engagement. Current and alumni members, as well as parents, reported a broad range of wellbeing benefits from being a TR14er [17]. The young people and parents were keen that others also experienced these benefits so promoted the group to others; recruiting new members, and seeking to secure the continuation of the Group after they left. For example, to ensure that future leadership was always in development a leadership programme had been created. Alongside formal processes to support succession planning the Group had shared terms of engagement that guided the relationships within the group. At the heart of these terms of engagement is a focus on the rights of children and young people that acts as an overarching paradigm for why the group exists and what it does. This focus on *Why* the Group exists that informs *What* it does has meant that the members feel able to adapt and evolve the dance sessions as their needs and interests shift and evolve. These processes could support the development of cultures of health in other locations [10,14–16,42].

The framework of 12 complex systems’ leverage points established by Meadows [15] starts with the importance of paradigms within complex adaptive social systems and having paradigms that transcend existing structures to reshape existing practice. The paradigm, goals and self-organisation adopted by the TR14ers (Table 1) aligns with Simon Sinek’s Golden Circle of *Why-How-What* [43]. Sinek’s Golden Circle is intended to help leaders develop successful and sustained teams; through first focusing on *Why* the team or organisation has been formed, then working out *How* to achieve that goal before finally determining *What* to do [43]. There are only a few other examples of Sinek’s work being applied in health research [44,45]. Both Aebersold, McCullagh [44] and Lam [45] describe the development of teams as part of health services or health research; we suggest that Sinek’s ideas can also be applied to community-based health promotion. However, much of public health commissioning and research funding is focused on *What* will be delivered and to whom; focusing on the process of delivery rather than the intention and core values (paradigm) of an intervention. Similarly, in respect to scaling up effective programmes of activities, Koorts and Rutter [42] have suggested that rather than trying to replicate an intervention (such as the TR14ers) by setting up dance groups in other areas, more success might be achieved by taking the central paradigm (the *Why*) of a successful intervention and working with the local community to co-create the *How* and *What* for that community.

Our new study contributes to the evidence supporting peer-led co-created health promotion interventions. The GoActive and Youth Engagement and Action for Health (YEAH!) programmes are other examples of co-created and peer-led physical activity interventions for young people [46,47]. GoActive was an iteratively developed 12-week intervention that trained older adolescents to be mentors and encourage younger classes to undertake additional physical activity [46]. However, it was not found to counter the age-related decline in physical activity [46]. YEAH! was a 10-week programme teaching adolescents from underserved communities in the USA, advocacy skills around community physical activity and nutrition assets [47]. Participants reported improvements in physical activity, nutrition and broader outcomes like peer support and confidence to influence adults [47].

One particularly novel aspect of how the TR14ers operate relates to information flows (Table 1). Co-ordinator 2 described how the TR14ers do not collect information about demographics or protected characteristics of members. This is a reflection of children’s rights being universal as the central paradigm of the TR14ers; consequently everyone is welcome, and you don’t need to meet some eligibility or representation criteria to participate. In turn, this means that the organisation is flatter with responsibility and power distributed among the members, with no small group of people holding sensitive information about members. A number of the alumni and parents talked about everyone taking care of each other (Table 1 and S4 Appendix). ‘It’s the whole, if one of us is failing then we are all failing because we haven’t picked up on it, so if one person is upset or stressed out or something then it falls on the rest of us to make sure that we pick up their slack.’ (Alumni 3)

The present study responded to the call from Greenhalgh and Papoutsi [10] for more mixed methods in-depth case studies of complex interventions. Further to this we recommend that studying existing long-standing interventions will provide insights into the mechanisms underpinning the creation of sustainable health promotion interventions. We employed two existing frameworks (Meadows [15] 12 complex systems’ leverage points and the five domains of adolescent wellbeing developed by the United Nations H6+ Technical Working Group on Adolescent Health and Well-Being [17]) to ground the case study of an existing community asset in current thinking about complex systems and adolescent wellbeing, and future studies may also find employing relevant frameworks helpful to building up the evidence base around complex interventions. However, some of the insights from the current study present a challenge to current service commissioning and research funding practices. Firstly, it is unlikely that the culture of health present in the TR14ers was there from day one, it has probably had to develop and evolve over time, which may have taken longer than a 1–3 year research grant or commissioning cycle. The process of deriving the *How* and *What* an intervention should be, developed from an agreed understanding and commitment to the *Why*, that also takes time and means that the intervention would be different in other places and communities and will change and evolve over time. Whereas, many public health interventions fail to be sustained once resources or circumstances change and hence mixed methods case studies of sustained activities are needed to understand the conditions by which community assets self-organise and are sustained.

The current case study was a small and short term piece of developmental research with a number of limitations and strengths. Firstly, the study was not designed or powered to test the effectiveness of the TR14ers and subsequently the assessment of the impact of the TR14ers is based on cross-sectional data and reports from participants in the study. The qualitative data were collected from a relatively small convenience sample of adults still involved or in contact with the Group. We could not recruit people who had joined and left the TR14ers for negative reasons or young people who did not want to join. However, we did assess the credibility of the findings from the interviews through the co-development workshops with young people who were currently part of the Group. Within Table 1 and S4 Appendix we have presented a variety of quotes from the participants demonstrating the variation in experience; no counter perspectives emerged during the interviews, possibly because of our convenience sample. There was some discussion about how one of the Co-ordinators was critical to the success of the TR14ers, ‘a community champion’, but that individual spoke in their interview about finding people to replace them. The scope and scale of this study mean that the findings should not be considered definitive and further studies using diverse methods in other long-standing community interventions are needed to test the findings of this study.

## Conclusion

The self-organisation of community members around the paradigm of universal children’s rights appears to have enabled the TR14ers to adapt and thrive for almost two decades contributing to child and adolescent wellbeing in an economically disadvantaged area in West Cornwall, UK. An in-depth mixed methods approach was necessary to understand how the Group attracts and keeps members. Interventions seeking to improve the health of the public should identify the paradigms that will reshape systems to create cultures of health. However, this is likely to mean that the intervention will evolve over time making it less predictable but more sustainable. Having participants who want to encourage others to join an intervention is a sign of the success of the intervention and is likely to contribute to its longevity. However, these attributes that are critical to the success and longevity of the TR14ers make community assets like this more challenging to fund or commission.

## Supporting information

S1 Appendix TR14ers dance leader’s contract. (PDF)

S2 Appendix Topic guide for parent/carer interviews. (PDF)

S3 Appendix Preliminary logic model. (PDF)

S4 Appendix Evidence of the TR14ers meeting the five domains of adolescent wellbeing [17]. (PDF)

S5 Appendix Poster on display during TR14er workshops. (PDF)

S6 Appendix SRQR checklist for TR14ers paper. (PDF)

S7 Appendix TIDieR checklist for TR14ers paper. (PDF)

S8 Appendix STROBE checklist for TR14ers paper. (PDF)
