# Supplementary material for: A complexity-informed in-depth case study into the sustainability and impact of a culture of health: The TR14ers community youth dance group
Source: PLoS One. 2023 Oct 25;18(10):e0293274. doi: 10.1371/journal.pone.0293274 (PMC10599586; doi:10.1371/journal.pone.0293274)
Supplement: S1 Appendix — (PDF) [file pone.0293274.s001.pdf]

S1 Appendix. TR14ers Dance Leader's contract

TR14ers Dance Leader's Contract

This contract is to make clear what is expected of Leaders, so everyone knows where they stand. It has been put together by the leaders to ensure everyone had a say in what was included.

To be a Leader this is what is expected of you. If you are unable to follow the things listed below, there will be 2 chances to correct it, if it continues after that we will have to take away your Leader status.

As a TR14ers Leader you will: -

- Help, support and advise members
- Show continual respect to everyone
- Support each other and ensure every single person feels valued and appreciated
- Co-operate with everyone
- Let [a Co-ordinator] know if you will be late or are unable to attend ideally 24 hrs prior to the session, but no later than lunchtime on the Friday.
- Not swear or bully anyone
- Choreograph your own dance routines
- Be able to teach, building towards being able to teach
- Not mess about
- Keep your energy positive and upbeat
- Lead by example
- Spread the word about TR14ers via social media and Word Of Mouth

If you have any concerns or worries at all, please speak to [a Co-ordinator].

Remember, you guys rock!!!! This contract is here to help you. It makes it clear what is expected of you and gives you guidelines, so you are all doing the same thing, and everyone is equal.

If you agree to the above, please sign below.

Name:

Signature: Date:
