# Supplementary material for: A complexity-informed in-depth case study into the sustainability and impact of a culture of health: The TR14ers community youth dance group
Source: PLoS One. 2023 Oct 25;18(10):e0293274. doi: 10.1371/journal.pone.0293274 (PMC10599586; doi:10.1371/journal.pone.0293274)
Supplement: S2 Appendix — (PDF) [file pone.0293274.s002.pdf]

S2 Appendix. Topic guide for parent/carer interviews

Topics to be covered include:

Background:
- Please can you tell me a bit about yourself and your family?
- Do you live in Camborne?
- Which school does your child go to?
- How did you hear about the TR14ers?

About the TR14ers:
- Can I ask you a bit about your child's participation with the TR14ers- how long has your child been coming here?
- What were your first impressions of the Group?
    - Have they changed?
- How would you describe the Group to a friend/ neighbour?
- Does TR14ers feel different to any other groups your child is part of and if so how?

Impact of TR14ers:
- Do you think being part of the Group has had an impact on your child?
    - If so what and when did you notice?
- Do you think being part of the Group has had an impact on you as a family?
    - If so what and when did this start to happen
- We are hoping to understand what effect being part of the Group has on young people's health so that we can support other young people to be as healthy as possible. We will be looking at their activity levels and asking them some general health questions but are there other things you think we should be looking at?
    - e.g. confidence, learning at school/ school results
