# Supplementary material for: A complexity-informed in-depth case study into the sustainability and impact of a culture of health: The TR14ers community youth dance group
Source: PLoS One. 2023 Oct 25;18(10):e0293274. doi: 10.1371/journal.pone.0293274 (PMC10599586; doi:10.1371/journal.pone.0293274)
Supplement: S4 Appendix — (PDF) [file pone.0293274.s004.pdf]

S4 Appendix. Evidence of the TR14ers meeting the five domains of adolescent wellbeing [17]

Domain	Subdomains	TR14ers evidence
Good health and optimum nutrition	• Physical health and capacities<br>• Mental health and capacities<br>• Optimal nutritional status and diet	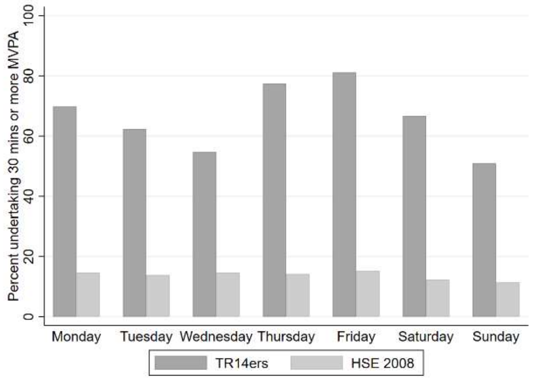<br>Participant moderate to vigorous physical activity (MVPA) compared to nationally representative data from the 2008 Health Survey for England (HSE) [30].<br>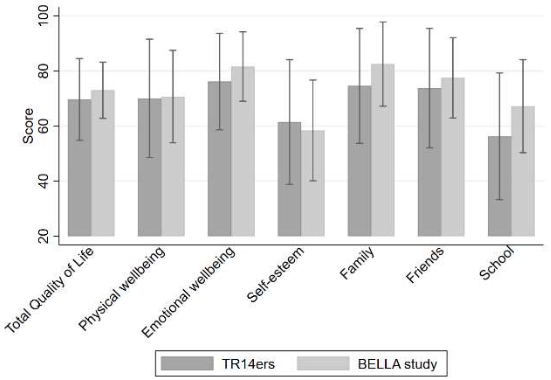<br>Participant health and wellbeing scores compared to the BELLA study* using the self-reported KINDL instruments [28].<br><br>'And from doing dance myself, there were times when I would come and I'd be in a foul mood because this and that happened. I would leave dance in such a better mood, because you can just get it out, whether it's by dancing or contemporary dancing, stuff where you can genuinely put feeling into it. And you go out feeling better.' (Alumni 4)<br><br>'Physically, I think it's amazing because, yes, they are exercising. Their lives are quite sedentary just because of the modern world we are in with TVs and screens and not being outside very much. So I really like that, yes, they are physical, and they kind of take that home as well, they practice at home, pushing themselves a bit at home as well and it has definitely impacted both of them in terms of confidence and just the experience of being part of a group and teaching the Group.' (Parent 6)
Connectedness, positive values,	• Connectedness: Is part of positive social and cultural networks and	'Everybody mixes together, so you've got a social part of it. You've got the learning of dancing, so that's the little bit of discipline that's in it; you've got the exercise, so it could be

and contribution to society	has positive, meaningful relationships with others, including family, peers, and, where relevant, teachers and employers<br>• Valued and respected by others and accepted as part of the community.<br>• Attitudes: Responsible, caring, and has respect for others. Has a sense of ethics, integrity, and morality.<br>• Interpersonal skills: Empathy, friendship skills, and sensitivity<br>• Activity: Socially, culturally, and civically active<br>• Change and development: Equipped to contribute to change and development in their own lives and/or in their communities	like an exercise club, you've got that side of it. There's lots of different things. And then you've got the adults on the outside that help run it and you see sometimes children might be upset and they are quite happy to go over and chat, open up and talk away to the adults. Maybe a child at home can't talk to their parents, might feel happy that somebody at dance is there that they can go and chat to, so you've got that side of it as well.' (Parent 2)<br><br>'TR14ers is like a young people's charity run by young people, so they give you the responsibility. They give you the chances and opportunities to lead, to learn, and if you want to learn, you can, like it's totally up to you. If you don't want to learn they are still there for you. But they'll give you something else to keep you involved and to make you feel like you're still getting the most out of it.' (Alumni 2)<br><br>'When I first came in I thought it was amazing. I couldn't believe how many children came. I was thinking maybe 10, 12 and I came in and it was just like a hall full of all ages and I thought wow, this is brilliant. And all the music blaring out, and the children just dancing and it doesn't matter if they can't do it, they can do their own little crazy dance. I just thought it was absolutely brilliant, really good, and it's nice, they get some of the younger ones to go in front and teach. I just think it's real confidence builder, it's a good social thing, if one child isn't good at socialising it doesn't matter, because you're put into little groups and things and everyone just starts chatting, so I think it really brings people out of themselves.' (Parent 2)
Safety and a supportive environment	• Safety: Emotional and physical safety<br>• Material conditions in the physical environment are met<br>• Equity: Treated fairly and have an equal chance in life<br>• Equality: Equal distribution of power, resources, rights, and opportunities for all<br>• Non-discrimination<br>• Privacy	'It kind of felt like a safe place, it was like a second home type of thing and when I got comfortable with it, it was like the only thing I would look forward to was going to TRs every week and that literally was the only thing that would keep me going was knowing that I would go to TRs on a Friday.' … 'TRs just felt like a safe place, like no matter what happened at school or anything like that, I just knew that I could go to TRs.' (Alumni 5)<br><br>'It's probably easier for us to actually see where people need help or if some people are just sort of flagging a bit because, like you said, we've been there ourselves, so we can recognise when people are just in a bad mood or if there's something wrong.' (Alumni 3)<br><br>'I would keep everyone together and there's got to be a core ethos that every single person in that room is equal to the other; there's no-one better than the other. And that is what, I

	• Responsive: Enriching the opportunities available to the adolescent	think, builds the confidence. Because it's terrifying, standing in front of a group of... Sometimes now there are 50 kids and you've got someone who's nine, teaching 50 kids, or young people should I say, not kids, right up to the age of 19, 20. And they are doing it and everyone is engaged.' (Co-ordinator 1)<br><br>'He feels safe. Nobody's judging him. He can go over and be with the older leaders and be part of the Group. They don't say, go away you're little. They just encourage him in. He feels he's part of the family, he's blossomed into a leader. Even his father was like, he wants to what?! Does he know what that (leader) means?!' (Parent 1)
Learning, competence, education, skills, and employability	• Learning: Has the commitment to, and motivation for, continual learning<br>• Education<br>• Resources, life skills, and competencies: Has the necessary cognitive, social, creative, and emotional resources, skills (life/decision-making) and competencies to thrive, including knowing their rights and how to claim them, and how to plan and make choices<br>• Skills: Acquisition of technical, vocational, business, and creative skills to be able to take advantage of current or future economic, cultural, and social opportunity<br>• Employability<br>• Confidence that they can do things well	'Role modelling the coordinator "I could watch how she talks to people, how she leads people and I've been able to speak to parents' kids, adults that I used to go really shy at or people my age, I'd back away from and let someone else talk for a while." "She's been a massive, massive influence on my confidence."' (Alumni 2)<br><br>'And it's not even just for dancing, it's a lot of the things that you can cross over into everyday life like being able to control the room helps you with your confidence or being essentially a team leader, so it gives you those leadership skills. It also gives you the confidence to be able to hold yourself and be assertive when you need to and it also gives you the skills for basic memory recall and stuff like that.' (Alumni 3)<br><br>'And, to be fair, two years ago if you were to have said to me, "Do you want to go to university?" I'd have laughed at you. I really would have laughed at you and I'd be like, "No chance." But no, now I am.' (Alumni 4)<br><br>'Being head doorman, for example, as a job. My job involves teaching. And I didn't teach until I came here.' (Alumni 4)

Agency and resilience	• Agency: Has self-esteem, a sense of agency and of being empowered to make meaningful choices and to influence their social, political, and material environment and has the capacity for self-expression and self-direction appropriate to their evolving capacities and stage of development<br>• Identity: Feels comfortable in their own self and with their identity(s), including their physical, cultural, social, sexual, and gender identity<br>• Purpose: Has a sense of purpose, desire to succeed, and optimism about the future<br>• Resilience: Equipped to handle adversities both now and in the future, in a way that is appropriate to their evolving capacities and stage of development<br>• Fulfilment: Feels that they are fulfilling their potential now and that they will be able to do so in the future	'…in order to get young people to teach young people, you first have to inspire those young people.' (Co-ordinator 1)

* Ravens-Sieberer U, Erhart M, Wille N, Bullinger M and the Bella study group. Health-related quality of life in children and adolescents in Germany: results of the BELLA study. *Eur Child Adolesc Psychiatry*. 2008;17(1):148-56. doi: 10.1007/s00787-008-1016-x.
