# Supplementary material for: A complexity-informed in-depth case study into the sustainability and impact of a culture of health: The TR14ers community youth dance group
Source: PLoS One. 2023 Oct 25;18(10):e0293274. doi: 10.1371/journal.pone.0293274 (PMC10599586; doi:10.1371/journal.pone.0293274)

S5 Appendix. Poster on display during TR14er workshops

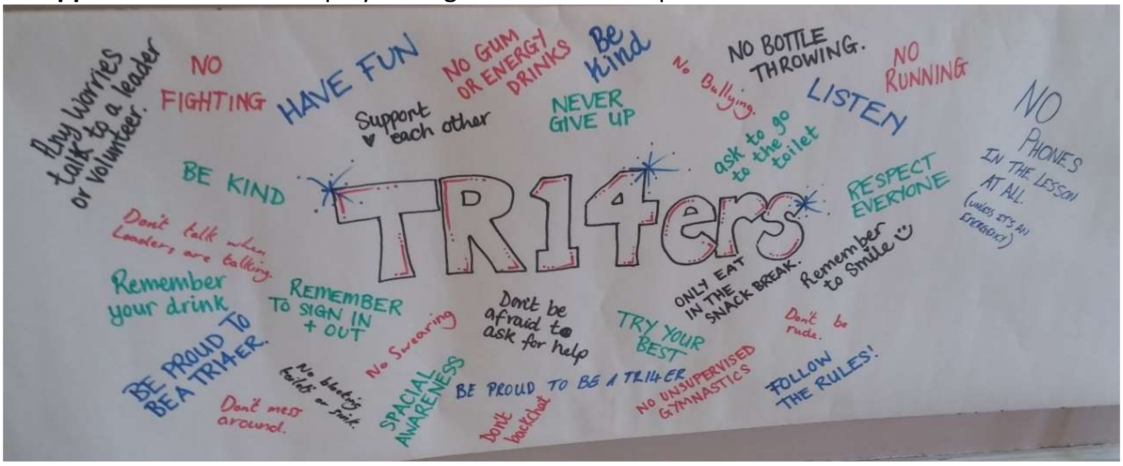

Supplement: S5 Appendix — (PDF) [file pone.0293274.s005.pdf]
