# Supplementary material for: A complexity-informed in-depth case study into the sustainability and impact of a culture of health: The TR14ers community youth dance group
Source: PLoS One. 2023 Oct 25;18(10):e0293274. doi: 10.1371/journal.pone.0293274 (PMC10599586; doi:10.1371/journal.pone.0293274)
Supplement: S6 Appendix — (PDF) [file pone.0293274.s006.pdf]

No.	Topic	Location in text	Item
	**Title and abstract**		
S1	Title	Lines 1-2	Concise description of the nature and topic of the study Identifying the study as qualitative or indicating the approach (e.g., ethnography, grounded theory) or data collection methods (e.g., interview, focus group) is recommended
S2	Abstract	Lines 17-37	Summary of key elements of the study using the abstract format of the intended publication; typically includes background, purpose, methods, results, and conclusions
	**Introduction**		
S3	Problem formulation	Lines 39-62	Description and significance of the problem/phenomenon studied; review of relevant theory and empirical work; problem statement
S4	Purpose or research question	Lines 64-81	Purpose of the study and specific objectives or questions
	**Methods**		
S5	Qualitative approach and research paradigm	Lines 157-179 and 196-224	Qualitative approach (e.g., ethnography, grounded theory, case study, phenomenology, narrative research) and guiding theory if appropriate; identifying the research paradigm (e.g., postpositivist, constructivist/ interpretivist) is also recommended; rationale[b]
S6	Researcher characteristics and reflexivity	Lines 270-279	Researchers' characteristics that may influence the research, including personal attributes, qualifications/experience, relationship with participants, assumptions, and/or presuppositions; potential or actual interaction between researchers' characteristics and the research questions, approach, methods, results, and/or transferability
S7	Context	Lines 108-149	Setting/site and salient contextual factors; rationale[b]
S8	Sampling strategy	Lines 180-211	How and why research participants, documents, or events were selected; criteria for deciding when no further sampling was necessary (e.g., sampling saturation); rationale[b]
S9	Ethical issues pertaining to human subjects	Lines 180-193	Documentation of approval by an appropriate ethics review board and participant consent, or explanation for lack thereof; other confidentiality and data security issues
S10	Data collection methods	Lines 196-267	Types of data collected; details of data collection procedures including (as appropriate) start and stop dates of data collection and analysis, iterative process, triangulation of sources/methods, and modification of procedures in response to evolving study findings; rationale[b]
S11	Data collection instruments and technologies	S3 Appendix	Description of instruments (e.g., interview guides, questionnaires) and devices (e.g., audio recorders) used for data collection; if/how the instrument(s) changed over the course of the study
S12	Units of study	Lines 282-307 and Fig 1	Number and relevant characteristics of participants, documents, or events included in the study; level of participation (could be reported in results)
S13	Data processing	Lines 196-224	Methods for processing data prior to and during analysis, including transcription, data entry, data management and security, verification of data integrity, data coding, and anonymization/deidentification of excerpts
S14	Data analysis	Lines 196-224	Process by which inferences, themes, etc., were identified and developed, including the researchers involved in data analysis; usually references a specific paradigm or approach; rationale [b]
S15	Techniques to enhance trustworthiness	Lines 196-224	Techniques to enhance trustworthiness and credibility of data analysis (e.g., member checking, audit trail, triangulation); rationale[b]
	**Results/findings**		
S16	Synthesis and interpretation	Lines 312-404	Main findings (e.g., interpretations, inferences, and themes); might include development of a theory or model, or integration with prior research or theory
S17	Links to empirical data	Lines 312-404	Evidence (e.g., quotes, field notes, text excerpts, photographs) to substantiate analytic findings
	**Discussion**		
S18	Integration with prior work, implications, transferability, and contribution(s) to the field	Lines 407-477	Short summary of main findings; explanation of how findings and conclusions connect to, support, elaborate on, or challenge conclusions of earlier scholarship; discussion of scope of application/ generalizability; identification of unique contribution(s) to scholarship in a discipline or field
S19	Limitations	Lines 478-493	Trustworthiness and limitations of findings
	**Other**		
S20	Conflicts of interest	Line 517	Potential sources of influence or perceived influence on study conduct and conclusions; how these were managed
S21	Funding	Funding statement	Sources of funding and other support; role of funders in data collection, interpretation, and reporting

[a]The authors created the SRQR by searching the literature to identify guidelines, reporting standards, and critical appraisal criteria for qualitative research; reviewing the reference lists of retrieved sources; and contacting experts to gain feedback. The SRQR aims to improve the transparency of all aspects of qualitative research by providing clear standards for reporting qualitative research.

[b]The rationale should briefly discuss the justification for choosing that theory, approach, method, or technique rather than other options available, the assumptions and limitations implicit in those choices, and how those choices influence study conclusions and transferability. As appropriate, the rationale for several items might be discussed together.
