# Supplementary material for: A complexity-informed in-depth case study into the sustainability and impact of a culture of health: The TR14ers community youth dance group
Source: PLoS One. 2023 Oct 25;18(10):e0293274. doi: 10.1371/journal.pone.0293274 (PMC10599586; doi:10.1371/journal.pone.0293274)
Supplement: S8 Appendix — (PDF) [file pone.0293274.s008.pdf]

STROBE Statement—Checklist of items that should be included in reports of ***cross-sectional studies***

	Item No	Recommendation	Location in manuscript
**Title and abstract**	1	(*a*) Indicate the study's design with a commonly used term in the title or the abstract	Lines 1-4
		(*b*) Provide in the abstract an informative and balanced summary of what was done and what was found	Lines 17-37
**Introduction**			
Background/rationale	2	Explain the scientific background and rationale for the investigation being reported	Lines 39-106
Objectives	3	State specific objectives, including any prespecified hypotheses	Lines 64-81
**Methods**			
Study design	4	Present key elements of study design early in the paper	Lines 156-193
Setting	5	Describe the setting, locations, and relevant dates, including periods of recruitment, exposure, follow-up, and data collection	Lines 180-181
Participants	6	(*a*) Give the eligibility criteria, and the sources and methods of selection of participants	Lines 182-193
Variables	7	Clearly define all outcomes, exposures, predictors, potential confounders, and effect modifiers. Give diagnostic criteria, if applicable	Lines 226-249
Data sources/ measurement	8*	For each variable of interest, give sources of data and details of methods of assessment (measurement). Describe comparability of assessment methods if there is more than one group	Lines 226-249
Bias	9	Describe any efforts to address potential sources of bias	NA – feasibility study
Study size	10	Explain how the study size was arrived at	Fig 1
Quantitative variables	11	Explain how quantitative variables were handled in the analyses. If applicable, describe which groupings were chosen and why	Lines 226-249
Statistical methods	12	(*a*) Describe all statistical methods, including those used to control for confounding	Lines 226-249
		(*b*) Describe any methods used to examine subgroups and interactions	NA
		(*c*) Explain how missing data were addressed	NA – feasibility study
		(*d*) If applicable, describe analytical methods taking account of sampling strategy	NA – feasibility study
		(*e*) Describe any sensitivity analyses	NA – feasibility study
**Results**			
Participants	13*	(a) Report numbers of individuals at each stage of study—eg numbers potentially eligible, examined for eligibility, confirmed eligible, included in the study, completing follow-up, and analysed	Fig 1
		(b) Give reasons for non-participation at each stage	Fig 1

		(c) Consider use of a flow diagram	
Descriptive data	14*	(a) Give characteristics of study participants (eg demographic, clinical, social) and information on exposures and potential confounders	Lines 282-294
		(b) Indicate number of participants with missing data for each variable of interest	Fig 1
Outcome data	15*	Report numbers of outcome events or summary measures	Lines 312-330
Main results	16	(*a*) Give unadjusted estimates and, if applicable, confounder-adjusted estimates and their precision (eg, 95% confidence interval). Make clear which confounders were adjusted for and why they were included	NA
		(*b*) Report category boundaries when continuous variables were categorized	NA
		(*c*) If relevant, consider translating estimates of relative risk into absolute risk for a meaningful time period	NA
Other analyses	17	Report other analyses done—eg analyses of subgroups and interactions, and sensitivity analyses	NA
**Discussion**			
Key results	18	Summarise key results with reference to study objectives	Lines 407-421
Limitations	19	Discuss limitations of the study, taking into account sources of potential bias or imprecision. Discuss both direction and magnitude of any potential bias	Lines 478-493
Interpretation	20	Give a cautious overall interpretation of results considering objectives, limitations, multiplicity of analyses, results from similar studies, and other relevant evidence	Lines 496-506
Generalisability	21	Discuss the generalisability (external validity) of the study results	Lines 422-477
**Other information**			
Funding	22	Give the source of funding and the role of the funders for the present study and, if applicable, for the original study on which the present article is based	Funding statement

*Give information separately for exposed and unexposed groups.

Note: An Explanation and Elaboration article discusses each checklist item and gives methodological background and published examples of transparent reporting. The STROBE checklist is best used in conjunction with this article (freely available on the Web sites of PLoS Medicine at http://www.plosmedicine.org/, Annals of Internal Medicine at http://www.annals.org/, and Epidemiology at http://www.epidem.com/). Information on the STROBE Initiative is available at www.strobe-statement.org.
